# The Corona-Pandemic: A Game-Theoretic Perspective on Regional and Global Governance

**DOI:** 10.1007/s10640-020-00475-4

**Published:** 2020-08-04

**Authors:** Alejandro Caparrós, Michael Finus

**Affiliations:** 1 Institute for Public Goods and Policies, Spanish National Research Council (CSIC), 28037 Madrid, Spain; 2 Department of Economics, University of Graz, Graz, Austria; 3 Department of Economics, University of Bath, Bath, UK

**Keywords:** Weakest-link game, Game theory, Experiments, COVID-19, Coordination, Cooperation

## Abstract

We argue that the incentive structure of all individual and coordinated measures across countries to contain the corona-pandemic is that of a weakest-link public good game. We discuss a selection of theoretical and experimental key results of weakest-link games and interpret them in the light of the corona-pandemic. First, we highlight that experimental evidence does not support the assumption that coordination can be trivially solved, even among symmetric players. Second, we argue that for asymmetric countries the weakest-link game does not only pose a problem of coordination, but also a problem of cooperation. Third, we show how and under which conditions self-enforcing treaties can foster coordination and cooperation. We account for the possibility that countries make mistakes when choosing their actions. Our discussion shows that North–South cooperation is relevant and likely to be self-enforcing and that regional cooperation, e.g., within the EU, will also be important.

## Introduction

The world is facing the worst pandemic in a century,^1^ caused by a new form of coronavirus.^2^ In a highly interconnected world, efforts to mitigate the effects of COVID-19 need to be coordinated, as an outbreak anywhere in the world puts all other countries at risk.^3^ That is, if one country relaxes its control measures and provokes an outbreak, all other countries will be negatively affected. The same logic applies to regions within a country, or states in the US. In addition, even wealthy and developed countries in Europe have seen that individual efforts may not suffice to control an outbreak. The situation will be even worse if serious outbreaks occur in developing countries or countries in transition, like currently observed for instance in Brazil and India. This implies that cooperation, and not only coordination, is needed to address this pandemic.

Ideally, one would look at previous experiences to learn lessons on how to react to such a pandemic. However, serious pandemics are rare events and there is simply not enough evidence to draw statistically significant conclusions. Furthermore, COVID-19 may very well be the first serious pandemic in a truly interconnected world. Hence, theory and experimental results provide the best guidance available. For climate change, theory and experiments predicted that the burden-sharing approach followed in the Kyoto Protocol was not an effective strategy. It took the world almost 20 years to realize this and to change this approach. We may not have the luxury of such a delay in finding the right strategy to tackle COVID-19.

We argue below that the relevant incentive structure is that of a weakest-link public good game (Hirshleifer 1983): the contribution of the agent that contributes the least determines the outcome of all.^4^ In the context of the eradication of smallpox and other infectious diseases, Barrett (2016) argued that the incentives are best described by such a game. He focuses on a static (one shot) game in pure strategies with symmetric countries (all countries have the same actions and payoff functions) where every country chooses a vaccination level with the knowledge that there exists a critical vaccination level, above which the disease is eliminated within the country (see also Barrett 2003; Barrett and Hoel 2007).^5^ Instead of focusing on eradication, we argue that, in the context of the COVID-19, weakest-link type incentives go beyond the eradication of the disease. Controlling outbreaks has this feature (e.g., social distancing, cellphone apps that trace contacts and the spread of the virus), individual decisions to wear a mask have this feature and providing a vaccine to all will eventually have this feature. In addition, given that there is no vaccine currently available, and that there are large uncertainties about the date at which such a vaccine will be available (if ever), it is too early to focus on eradication. Thus, an abstract weakest-link game is more relevant, as it is able to capture the incentives not only for eradication, but also for control and prevention measures. In such a framework, we show that coordination on the best possible outcome is far from trivial in a weakest-link game (even assuming symmetric players). In fact, abandoning the assumption that no country makes mistakes (i.e., introducing some realism into the analysis), a failure of coordination, implying the worst possible outcome in the game, is individually rational and completely in line with experimental results.

The analysis in Barrett (2016) concludes on a rather positive note, as smallpox was eradicated. However, as already discussed in Barrett (2003), there is no guarantee that this result will hold for all diseases. Therefore, Barrett (2003) already discusses the usefulness of (cooperative) institutions to coordinate on eradication. This may be viewed as “cooperation to coordinate” on one of the individually rational equilibria for symmetric players. We will show below that, once asymmetric countries are introduced in the analysis, this type of cooperation is not sufficient: additional cooperative efforts in the form of transfers are needed to achieve the first-best outcome. Fortunately, when cooperative institutions are introduced, all the different analytical frameworks (theory and experiments) discussed below support the idea that cooperation has a role to play in bringing countries to the first-best outcome in a perfect world, or at least closer to the first-best outcome in a less than perfect world. Moreover, the incentive structure in the context of the corona-pandemic appears to encourage successful self-enforcing cooperation.

There has been extensive research on the incentives of cooperation in weakest-link games, both theoretical (see the papers cited in Caparrós and Finus 2020) and experimental (see the papers cited in Devetag and Ortmann 2007). However, our goal is not to review this vast literature. Instead, we focus on some key results which we have obtained in previous research and interpret them in the light of the corona-pandemic. The remainder of this article is organized as follows. Section 2 presents the weakest-link game in the absence of treaties. First, theoretical results obtained in the absence of mistakes are discussed. Then, experimental results are presented that are at odds with those theoretical results. The last sub-section in Sect. 2 shows that behavioral theory, based on the assumption that agents may make (small) mistakes, is able to reconcile theoretical and experimental results. As Sect. 2 essentially rationalizes the failure of coordination in the absence of treaties, as observed in the lab, Sect. 3 discusses possible solutions, focusing on the role that treaties might have in fostering cooperation. Finally, Sect. 4 concludes and highlights relevant research gaps.

## The Problem: Coordination Failure

### Non-cooperative Theory Without Mistakes

Let the payoff function of player $i \in N$ be given by

1 $$\begin{aligned} \varPi_{i} (Q,\;q_{i} ) & = B_{i} (Q) - C_{i} (q_{i} ) \\ Q & = \mathop {\text{min} }\limits_{i \in N} \{ q_{i} \} \\ \end{aligned}$$

where $Q$ denotes the public good provision level, which is the minimum provision level over all players and $q_{i}$ denotes the individual provision level of player $i$. In our context, the public good is the intensity and quality of virus control.^6^ Benefits $B_{i} (Q)$ depend on the smallest contribution and costs, $C_{i} (q_{i} )$, depend on the individual contribution of player $i$. In a general theoretical setting, benefits are typically assumed to be concave and costs strictly convex such that the payoff function is strictly concave with a unique interior maximum from player $i$’s perspective. This maximum, which we denote by $q_{i}^{A}$, can be viewed as the optimal provision level under autarky (i.e., $B_{i}^{'} (q_{i}^{A} ) = C_{i}^{'} (q_{i}^{A} )$). Generally, if benefit and cost functions are different, autarky provision levels will be most likely different. Hence, we have $q_{{\text{min}}}^{A} = q_{1}^{A} \le q_{2}^{A} \le \cdots \le q_{n}^{A}$, indexing players according to their autarky levels.

If we denote by $Q_{ - i,\; {\text{min}} }$ the minimum provision level of all players except $i$, then the optimal response of player $i$ is to match $Q_{ - i,\;{\text{min}} }$ as long as $Q_{ - i,\;{\text{min}} } \le q_{i}^{A}$. As payoffs are concave, any provision level below $Q_{ - i,\;{\text{min}} }$ would imply a lower payoff to player $i$, and the same is true for any provision level above $Q_{ - i,\;{\text{min}} }$, as this would entail only additional costs but no additional benefits. For any level $Q_{ - i,\;{\text{min}} }$ above player $i$’s autarky level, i.e., $Q_{ - i,\;{\text{min}} } > q_{i}^{A}$, player $i$ will stick to his/her autarky level as player $i$ cannot be forced to provide more than $q_{i}^{A}$. Hence, player $i$ matches all provision levels up to level $q_{i}^{A}$. Thus, all provision levels with $q_{i} = q_{j}$ for all $i,j \in N$ from zero up to $q_{\text{min} }^{A}$ are Nash equilibria. Given the concavity of players’ payoff functions, the Pareto-optimal Nash equilibrium is obtained when all players match $q_{\text{min}}^{A}$, $q_{\text{min}}^{A} = q^{*} = q_{i}^{*} = q_{j}^{*}$ for all $i,j \in N$ and $i \ne j$ (Cornes 1993; Cornes and Hartley 2007; Vicary 1990; Vicary and Sandler 2002).

If all players have the same benefit and cost function, then $q_{i}^{A} = q_{j}^{A}$ for all $i,\;j \in N$, $i \ne j$. In this particular case, the Pareto-optimal Nash equilibrium is identical to the social optimum. Hence, in the absence of any further complication, there is no need for cooperation, only coordination is required to settle for the Pareto-optimal Nash equilibrium. This should prove easy as all interests are aligned. Interestingly, all experimental evidence of which we are aware proves this result wrong, as we report in Sect. 2.2. Before turning to this evidence, let us briefly discuss another important complication and feature of reality, namely asymmetry.

If players perceive benefits differently and face different costs, which is most likely the case in reality, then autarky provision levels will be different. In other words, players have different preference regarding the “optimal” provision level. Even if we make the heroic assumption that all players settle for the Pareto-optimal Nash equilibrium, this will no longer be socially optimal. As one can show that the socially optimal provision level, $$Q^{**}$$ with $$q_{i}^{**} = q_{j}^{**} = Q^{**}$$ for all $$i,\;j \in N$$, $$i \ne j$$ lies between the minimum and maximum autarky provision level over all players (Caparrós and Finus 2020), the difference between the socially optimal and the Pareto-optimal Nash equilibrium provision level can be quite large. Accordingly, coordination is no longer sufficient, and cooperation is needed in order to overcome or at least mitigate the loss of global welfare emerging if players behave non-cooperatively.

The COVID-19 has shown that inequalities matter. In Europe, countries with comparatively weaker sanitary systems and with social behavior with more proximity between people, have been hit hardest. As a result, respiratory equipment was shipped across countries and even patients were moved between countries. Furthermore, Europe is considering, for the first time, raising money by issuing bonds secured by all 27 members.^7^ This is a form of transfer, a monetary transfer in our abstract framework. At the global scale, inequalities are far starker, and cooperation will be even more critical. A formal analysis of the role of cooperation can be found in Sect. 3.

### Experimental Evidence

In experiments, it is important to keep things simple. Therefore, most papers assume a linear payoff function. A further simplification emerges if discrete provision levels are assumed. For instance, in Van Huyck et al. (1990) seven provision levels are considered, yielding the payoff matrix shown in Table 1. In their terminology, choosing a contribution level means choosing a number. In our context, this implies to summarize the different degrees of intensity and quality of virus control into a set of discrete actions.

**Table 1:** Payoffs in Van Huyck et al. (1990)

Number you choose	Smallest number chosen by any participant in your group (including yourself)						
	7	6	5	4	3	2	1
7	130	110	90	70	50	30	10
6		120	100	80	60	40	20
5			110	90	70	50	30
4				100	80	60	40
3					90	70	50
2						80	60
1							70

*Source* Van Huyck et al. (1990), Payoff Table A, p. 232, all entries multiplied by 100

In Table 1, the payoff which a player obtains for a given number depends on the smallest number chosen among all players, i.e., the minimum. For instance, if a player chooses the highest number 7, she will earn 130 if all players choose 7. However, if the lowest number chosen by others is 6, she will earn only 110. In our context, even if country *i* goes for the maximum possible level of virus control, if other countries do not follow the same strategy, there is a risk that COVID-19 will eventually affect country *i*, and this impact will be higher the smaller the number chosen by other countries.

All Nash equilibria lie on the diagonal in Table 1, and all players choosing 7 is the Pareto-optimal Nash equilibrium. In a given row, all entries to the right of the diagonal entry imply a lower payoff to player $i$, as other players choose a lower number than player $i$. The larger the difference between the own number and the minimum number of all others, the larger will be the loss. Thus, the risk of a loss increases with the number chosen by player *i*. Only if player $i$ chooses 1 she/he is sure to receive a payoff of 70.

Caparrós et al. (2020) run experiments for payoffs as given in Table 1. Their results are in line with Van Huyck et al. (1990), Feri et al. (2010) and many others (see Devetag and Ortmann 2007 for an overview). In experiments, typically, not only the minimum number, which determines the benefits of subjects, but also the average number is analyzed, which can be viewed as proxy of the average degree of coordination. The experimental results obtained in Caparrós et al. (2020) are as follows:

1. Average and minimum numbers decline with the number of rounds the weakest-link game is repeatedly played.
2. Average numbers approach values below 2, and minimum numbers approach quickly values close to 1 as the game is repeated.

Thus, there is no doubt, coordination is not as easy and straightforward as theory predicts, based on the assumption that players do not make mistake. Coordination on Pareto-superior outcomes does not emerge in the lab.

Several experimental studies have shown that this negative result can be alleviated, for example, by excluding neighbors (Riedl et al. 2006), introducing financial incentives (Brandts and Cooper 2006), reducing effort costs (Goeree and Holt 2005) or using advice by players who have played in previous rounds (Chaudhuri et al. 2009). In terms of their relevance for the COVID 19-crisis, excluding neighbors is probably the most relevant strategy. Riedl et al. (2016) show that when agents are given the possibility to exclude players from the group that share the weakest-link public good, the first-best outcome can be attained. Many countries, including the European Union as an entity have restricted or suspended travel from other countries. However, the question arises as to how effective such a strategy will be in the long-run in our interconnected world.^8^ Introducing financial incentives or reducing effort costs, appear only feasible within some form of institutionalized international cooperation to combat COVID-19. In order to be self-enforcing and sustainable over time, temporary and ad hoc cooperation may not be sufficient, appropriate international institutions are required, as discussed in Sect. 3. Advice from players who have played the game previously may have had a role at the beginning of the pandemic, as regions which were hit later could learn from regions where the virus emerged earlier. However, this learning seems to have been confined to national strategies. Learning in terms of international coordination and cooperation did not take place; just the opposite, many countries closed their borders and imposed an export restriction on health equipment and masks.

In a nutshell, the behavior of countries in terms of coordination and cooperation may be summarized as follows. Although European countries seem to have improved their coordination during the course of the crisis, at the peak of the pandemic, coordination was far from ideal, even prohibiting the export of medical equipment across EU countries. Regarding the near future, Trump has announced that the US, the largest contributor, will leave the WHO, imperiling future global efforts to provide vaccines for all.

Focusing on the experience with previous diseases provides mixed evidence. Coordination on smallpox eradication was relatively successful, probably because it had many attributes that facilitated coordination (as discussed in Barrett 2003). Type 2 and type 3 poliovirus have been declared eradicated, implying that two-thirds of wild polioviruses have been eradicated.^9^ However, efforts to eradicate other wild polioviruses continue, despite many years of efforts to eliminate them, mainly because of their weakest-link nature (Barrett 2007a). The very nature of the weakest-link game implies that even small disruptions can cause eradication to remain out of reach (Barrett 2016).

In addition, at least from the point of view of the developed world, smallpox implies a small degree of disruption of the economy. However, COVID-19 has affected the economy and the social structure of almost all countries around the globe to an (until recently) unimaginable degree.^10^ Thus, not only eradication but also mitigation of the impact of the virus is absolutely crucial for affected economies. To some extent, translating the success of coordination on smallpox to successful coordination (plus cooperation) on COVID-19 may be as difficult as translating the cooperation formula that was successful for the ozone layer (the Montreal Protocol) to a much more complex problem like climate change (Barrett 2007b).

### Non-cooperative Behavioral Theory with Mistakes

In order to explain the failure of coordination for symmetric payoffs in experiments, Caparrós et al. (2020) suggest to consider a quantal response equilibrium (QRE) which is a Nash equilibrium in mixed strategies based on a probabilistic choice function (McKelvey and Palfrey 1995). The idea is that decisions are stochastic, all actions have a positive probability and the probability of choosing non-optimal actions is inversely related to the possible loss. Hence, players assume that they and others may make mistakes, which may be very small, but with a non-zero probability; the probability of “costly errors” is lower than that of “cheap errors”. For a logit specification, the sensitivity of players to errors is measured by an “error parameter”$\lambda$. Larger values of $\lambda$ imply a larger sensitivity to errors and make non-optimal choices less likely. For $\lambda = 0$, choices are purely random and for $\lambda \to \infty$ choices are perfectly rational.

In order to provide an intuition for our experimental results discussed above, let us call $p_{xy}$ the probability that if player *i* selects *x* the minimum is *y*. For instance, in Table 1, the probability of receiving a payoff of 70 when choosing number 1 is one, $p_{11} = 1$. That is, this is a save choice. The probability $p_{22}$ of receiving a payoff of 80 when choosing number 2 is the probability that none of the seven other players chooses 1. The probability of receiving a payoff of 60 because at least one other player chooses 1 is therefore $1 - p_{22}$. The expected payoff when choosing 2 is therefore $p_{22} \cdot 80 + (1 - p_{22} ) \cdot 60$. When choosing higher numbers, it is clear that the probability that none of the other players chooses a lower number than player $i$ decreases exponentially. (That is, $p_{77}$ will be much smaller than $p_{22}$.) Hence, already for very low values of $\lambda$, the equilibrium yields a probability larger than 0.99 of playing 1. In other words, it is rational in a less than perfect world that players do not coordinate on the Pareto-optimal Nash equilibrium. In fact, it is (perfectly) rational to play save, with the consequence of the worst Nash equilibrium emerging. This explains why coordination is not trivial.

Mistakes are part of human behavior, and when confronted with a new problem, such as COVID-19, individuals, organizations and countries are likely to make mistakes. The WHO has modified its advice on several occasions, as new evidence came in (Chu et al. 2020). In addition, countries have failed to follow the advice of the WHO. Moreover, several countries, most notably the US, have criticized the organization for its management of the crisis.^11^ Individual countries have also modified their strategies: the UK modified its initial strategy (which was based on the objective of herd immunity) and many consider now that the lax approach followed by Sweden was probably a mistake.^12^ In the US, many states are reversing their course after rushing to reopen their economies.^13^ Hence, a theoretical framework that allows for mistake is probably very relevant for the analysis of the corona-pandemic. This implies that both, experiments and extended theory, predict that coordination will fail, resulting in the worst possible outcome. Given this somber prediction, the next section discusses possible ways out of this dilemma.

## Possible Solution: Treaties

### Cooperative Theory Without Mistakes

Let us now assume that players have the possibility to join a treaty and that players are asymmetric (as otherwise treaties are redundant in a world without mistake). Consider the simple cartel formation game, which may be regarded as the workhorse model used to analyze international environmental agreements.^14^ In the first stage, players decide whether to join a treaty or whether to remain an outsider. This leads to a coalition structure $C = \{ S,\;k,\; \ldots ,m\}$ with coalition $S$ and some singleton players if $S$ is not the grand coalition. In the second stage, players choose their contribution levels. The coalition is assumed to act as one player and hence its autarky provision level $q_{S}^{A}$ follows from the maximization of the aggregate payoff over all coalition members, which leads to the first order condition $\sum\nolimits_{i \in S} {B_{i}^{'} (q_{S}^{A} )} = \sum\nolimits_{i \in S} {C_{i}^{'} (q_{S}^{A} )}$. Non-members’ autarky provision levels $q_{k}^{A}$ are the same as without a treaty and follow from $B_{k}^{'} (q_{k}^{A} ) = C_{k}^{'} (q_{k}^{A} )$ as explained above. As before, all provision levels with $q_{S}^{{}} = q_{k} = q_{m}$ from zero up to the lowest autarky provision $q_{\text{min}}^{A}$, $q_{\text{min}}^{A} = {\text{min}} \{ q_{S}^{A} ,\;q_{k}^{A} ,\ldots,\;q_{m}^{A} \}$, are Nash equilibria, with the Pareto-optimal Nash equilibrium if coalition $S$ forms given by $q_{\text{min}}^{A} = q^{*} (S) = q_{S}^{*} = q_{k}^{*} = q_{m}^{*}$.

Suppose all players, including coalition $S$, are able to coordinate on this Pareto-optimal Nash equilibrium between coalition $S$ and all single players. Accordingly, payoffs are given by $\varPi_{1} (q^{*} (S)),\;\varPi_{2} (q^{*} (S)),\; \ldots ,\,\varPi_{n} (q^{*} (S))$. Moving back to stage 1, coalition $S$ is stable if

2 $$\text{internal}\,\text{stability:}\,\varPi_{i} \text{(}q^{*} \text{(}S\text{))} \ge \varPi_{i} \text{(}q^{*} \text{(}S\backslash {\{ }i{\} ))}\quad \forall \;i \in S$$

3 $$\text{external }\,\text{stability}:\,\varPi_{j} \text{(}q^{*} \text{(}S\text{))} \ge \varPi_{j} \text{(}q^{*} \text{(}S \cup \{ j\} \text{))}\quad \forall \;j \notin S$$

hold simultaneously. That is, no treaty-member should have an incentive to leave coalition $S$ (internal stability) and no non-member should have incentive to join coalition $S$ (external stability).

Clearly, coalition $S$ can only make a difference to no cooperation if $S$ includes the player with the smallest autarky provision level, which we denoted by $q_{1}^{A}$ above. (Recall player $1$ can veto any provision level above $q_{1}^{A}$) We call this an effective coalition. The equilibrium provision level of such an effective coalition $q^{*} (S)$ will be either the coalition’s autarky provision level $q_{S}^{A}$ or the smallest provision level among the outsiders $q_{k}^{A}$, which, in any case, implies that the equilibrium provision level is above player $1$’s autarky provision level, i.e., $q_{1}^{A} < q^{*} (S)$. Hence, player $1$’s payoff in coalition $S$ falls short of that under no cooperation when player $1$ determines the equilibrium, $q^{*} = q_{1}^{A}$. Since a coalition which is not profitable to all players is not internally stable, no effective coalition is stable in the absence of transfers.

With compensation payments among coalition members of the form

4 $$T_{i} = \alpha_{i} \sum\nolimits_{{k \in S\backslash {\{ }i{\} }}} {\sigma_{k} \text{(}S\text{)} - \text{(}1 - \alpha_{i} \text{)}} \sigma_{i} \text{(}S\text{)}\,\text{with}\,\sigma_{i} \text{(}S\text{)} = \varPi_{i} \text{(}q^{*} \text{(}S\text{))} - \varPi_{i} \text{(}q^{*} \text{(}S\backslash {\{ }i{\} ))}$$

each player in $S$ receives after transfer payments payoff $\varPi_{i}^{T} (q^{*} (S)) = \varPi_{i} (q^{*} (S\backslash \{ i\} ))$ $+ \alpha_{i} \sigma (s)$, with $\sigma (s) = \sum\nolimits_{i \in S} {\sigma_{i} (S)}$ called the total surplus of coalition $S$ and $\alpha_{i} > 0$ a share where shares add up to one, i.e., $\sum\nolimits_{i \in S} {\alpha_{i} } = 1$. Hence, each member receives after transfer payments his/her free-rider payoff $\varPi_{i} (q^{*} (S\backslash \{ i\} ))$ plus a share $\alpha_{i}$ of the total surplus $\sigma (s)$. The total surplus is the total payoff obtained by coalition $S$ minus the sum of free-rider payoffs of its members. Hence, if the total surplus $\sigma (s)$ is positive, we can conclude that coalition $S$ is internally stable if the transfer scheme in (4) is employed.^15^

The particular form of the transfer scheme in (4) implies that a coalition member $i$ receives a share $\alpha_{i}$ of the surplus generated by other coalition members and the second term captures the idea that member $i$ has to pay a share $(1 - \alpha_{i} )$ of the surplus that the coalition generates to player $i$.

Given the transfer scheme in (4), one can ask five questions (Caparrós and Finus 2020).

1. *Does an effective stable coalition exist?* The answer is yes, even though it is not possible to exactly predict which particular coalition is stable at such a general level. The intuition is that any two-player coalition, including the country with the smallest autarky provision level, is internally stable. The surplus is always strictly positive and a deviation will lead to the initial situation in which both countries are worse off. Such a coalition may or may not be externally stable. However, if it is not, then a larger coalition will eventually be internally and externally stable. Hence, cooperation on combating COVID-19 is generally feasible and self-enforcing.
2. *How does the distribution of autarky provision levels within coalition* $S$ *affect stability?* Distributions which are positively skewed favor stability. That is, distributions with autarky provision levels with many players having an autarky provision close to the average over all players in $S$ and one or two players with an autarky level well above this average are conducive to stability. Players with an autarky provision level well above the average pay net transfers to all players for staying on board. Thus, strong asymmetries are not an obstacle but an asset for stability. For combating COVID-19, this seems to favor cooperation in a classical North–South geopolitical context with large asymmetries between the two groups, with a much larger number of countries in the South.
3. *Can the grand coalition, i.e., the coalition including all players be stable?* The answer is affirmative. One can show that one outlier at the top is sufficient to ensure that the grand coalition is stable. The intuition is similar to what has been explained under 1) above. The surplus is positive and for such an extremely skewed distribution it happens that if one player leaves the agreement, all players are back to the situation with no agreement at all. Hence, the implicit punishment is sufficiently strong. Given that due to the transfer scheme all players receive a “fair” share of the surplus, no country has an incentive to leave the grand coalition. Again, extreme asymmetries are conducive to stability. Hence, in the context of COVID-19, even a global treaty could be potentially stable.
4. *How does stability and the gains from cooperation relate?* As a tendency, those distributions which favor stability imply low gains from cooperation and vice versa. This has some resemblance with the paradox of cooperation, which has been first established by Barrett (1994), and has been reiterated later several times in the context of public goods with a summation technology. Nevertheless, self-enforcing treaties under the weakest-link technology can make a difference to no cooperation. In the context of COVID-19, even though the gains from cooperation at the global scale may not appear to be huge, the gains at the level of individual developing countries may be still large and may save many lives. In particular, in the context of the weakest-link game, the theoretical results show that—different from public good games with a summation technology—it cannot be claimed that it is easier to form smaller than larger agreements, just the reverse may be true. In other words, there is no excuse to attempt only local or regional cooperation, global cooperation may prove to be easier and more effective.
5. *Will the qualitative conclusion also hold if players fail to perfectly coordinate on the Pareto*-*optimal Nash equilibrium in such a treaty game?* Again, the answer in Caparrós and Finus (2019) is affirmative, treaties can make a difference, though less than perfect coordination will entail a welfare loss. Thus, even in a less than perfect world, the qualitative conclusion that cooperation can make a difference, continues to hold. This nicely links theory with the experimental evidence on which we report below, although it is important to highlight that here we are referring to cooperating between asymmetric players (for COVID-19, think of North–South cooperation), while the experimental results presented below are valid only for cooperation between symmetric players (think of cooperation within the EU approximately).

In a less than perfect world, with large differences between countries, coordination is unlikely to be the sole answer and cooperation will be needed. Although a numerical analysis of whether or not the world is in a situation that favors the success of a treaty on cooperation in the Corona-weakest-link game is beyond the scope of this paper, we will informally discuss the likelihood of its success. What is needed for a successful self-enforcing treaty is basically a positively skewed distribution of autarky provision levels. This means a small group of strong countries, which are clearly stronger than the rest, and a large group of relatively weak countries. (In this context, ‘strong’ means to be interested in a high level of provision in autarky, i.e., without external support). Within the EU, this might not be the case. Hard hit countries include relatively poor countries (in the European context) such as Spain and Italy, but also relatively well-off countries such as France, Belgium and the UK. Furthermore, even relatively poor countries in Europe have excellent medical systems and would aim at a high provision level even under autarky. Hence, cooperation on this topic might prove to be more complicated than one would hope, focusing exclusively on the theoretical analysis in this subsection. (However, see the discussions of the experimental evidence in Sects. 3.2. and 3.3 for symmetric players.)

This would be different if the impacts on the economy turn out to be very unequal across Europe, impacting relatively poor countries much more. Hence, asymmetry could be an asset for the prospects of cooperation. Already before the pandemic, the European Commission proposed a European Green Deal (European Commission 2019, 2020a, b). The coronavirus has probably strengthened the interest of most countries in such a plan. The current initiative by the two “strong” players France and Germany for a 500 billion “rescue package” suggests that asymmetries, in particular in the long-term, may be larger than expected.

In any case, at a global level, the perspective for effective and self-enforcing cooperative efforts are certainly promising. Helm (2020) argues that the virus has reinforced the perceived importance and power of nation states over global institutions. However, one can also argue that the virus has shown countries how interconnected they are and that a national response may not suffice.^16^ Differences in the medical systems between developed and developing countries are stark, and this will probably imply large differences in protective measures (and vaccination efforts) between developed and developing countries under autarky. Furthermore, the number of developed (strong) countries is considerably smaller than the number of developing countries (weak) countries. This is especially true concerning measures to conduct mass vaccination. The number of countries able to provide the technology and the means to vaccinate at a massive scale is most likely small. Self-enforcing cooperation can be successful in this context, including transfers, as strong players have an interest in halting outbreaks in weak countries as they will ultimately reach their shores due to migration and tourism. This is true for COVID-19 but also for future pandemics. Hence, the world should work on establishing strong international institutions that facilitate cooperation on pandemic control.^17^ This is both necessary and not unlikely to succeed, given the prevailing incentive structure.

### Experimental Evidence

The theoretical analysis in Caparrós and Finus (2020) suggests that cooperation between symmetric (identical) countries is irrelevant for the weakest-link game (see also Sect. 2.1). In the COVID-19 context, this would imply that regional cooperation, such as cooperation within the EU, is not particularly relevant. However, the experimental evidence in Caparrós et al. (2020) suggests that even among symmetric players, cooperative institutions may substantially influence outcomes.

In Caparrós et al. (2020) the possibility of joining a treaty is called team formation. In the experiment, treatments are based on the payoffs in Table 1. The setting is similar to that in the cartel game (see Sect. 3.1), but with some important modifications. In fact, the degree of consensus in stage 1 and 2 is much higher than in the standard cartel formation game, as all decisions have to be taken by unanimity, which strengthens the ability of teams to reduce their risk. In stage 1, players can announce whether to join team $S$. This decision is disclosed to all players and those who have announced to join the team are asked to confirm their membership. If and only if all team members confirm their membership will the team form (otherwise it dissolves). In the second stage, provided a team has formed in the first stage, all members can propose a number of which the minimum number will automatically be selected. Single players choose their own number in this final stage. Thus, the assumed decision process in the experiment closely mimics the decision procedures within many international institutions, which very much rely on consensus. As it will turn out below, interestingly, consensus may not be an obstacle as commonly perceived but an asset for effective outcomes.

Clearly, in this setting, if the grand coalition forms, subjects face no risk by announcing the Pareto-optimal number 7. The risk of receiving a low payoff because an outsider chose a lower number than the coalition increases with the number of outsiders. The most important experimental results can be summarized as follows.

1. Subjects use the opportunity to form teams; they always initiate teams and confirm them in most (90% of the) cases.
2. Minimum numbers are higher with team formation than without team formation and downward trends over rounds may even be reversed. However, Pareto-optimal provision levels are not obtained.

Thus, in this setting, the opportunity of joining an agreement can contribute to addressing the coordination problem, but it does not solve it completely. When trying to explain insufficient coordination, a theory based on pure Nash equilibrium strategies, including the selection criteria of Pareto-dominance, has very limited predictive power for at least two reasons. First, there is still a multiplicity of equilibria. Second, insufficient coordination is not among the Pareto-undominated Nash equilibria of the game. In order to overcome this shortcoming, we introduce the possibility of mistakes (as before in Sect. 2.3) in the setting of cooperation.

### Behavioral Cooperative Theory with Mistakes

As mentioned above, one can rationalize these experimental results by applying the Quantal Response Equilibrium, though in an extended version,^18^ to capture the first and second stage of treaty formation. Whereas without team formation, even for low values of the rationality parameter $\lambda$, it was an equilibrium strategy to choose number 1, with team formation the opposite is true. For sufficiently high values of $\lambda$, it is an equilibrium strategy to join a team, in particular the grand team, and to choose minimum number 7. That is, rationality is a curse without team formation, but it is a blessing with the option to form teams. Obviously, in the experiment, subjects are not perfectly rational, which explains that the probability of joining the team is below one and minimum numbers are typically between 4 and 7, and the average minimum number is certainly below 7. Hence, team formation improves coordination but does not solve it entirely.

To sum up, experiments and behavioral theory, allowing for mistakes, resulted in a gloomy perspective in Sect. 2. Fortunately, once treaties (or other forms of institutionalized cooperation) are introduced, experiments and behavioral theory conclude that cooperative institutions play a positive and important role. This reinforces our arguments which we presented in the theory Sect. 3.1 about the positive and important role of institutionalized forms of international cooperation in order to combat the corona pandemic. In Sect. 3.1, we argued that international cooperation, in addition to coordination, is necessary to balance asymmetries between countries by designing optimal monetary transfers. For symmetric players, there was no reason for cooperation in a world of pure strategies and no mistakes. However, the experiments with symmetric players have shown that even under such “ideal conditions” coordination is anything else than trivial. Introducing a behavioral theory with mistakes explained this pessimistic result but also showed that cooperation can reverse those pessimistic conclusions. The upshot is that international cooperation will be relevant in the context of the COVID-19 pandemic between asymmetric counties but also between similar countries, as long as we presume that governments may make mistakes, which does not appear to be a very unrealistic assumption. Hence, building institutions which coordinate regional but also global cooperation will be important in the future in order to successfully combat COVID-19 and similar pandemics.

## Research Gaps and Concluding Remarks

In terms of research gaps, it is evident from Sects. 3 and 4 that one should extend experiments with and without team formation to asymmetric players, including the possibility of transfer payments within a team. This would add some realism.

Another item which is missing in theory and experiments is the possibility of in-kind transfers (i.e., non-monetary transfers). In a theoretical setting with asymmetric players but without the possibility of signing treaties it has been shown that in-kind transfers can raise the equilibrium provision level (Vicary and Sandler 2002). What is missing is to include in-kind transfer in the analysis of treaties. This is important because during the corona crisis countries received not only financial support, but also masks and technical equipment like ventilators, and patients were flown out to be treated in hospitals in other countries.

The results discussed above have shown that coordination tends to collapse in the lab in the weakest-link game and that theory supports this result, when possible mistakes are taken into account. This implies a somber perspective for coordination on the corona-pandemic. Coordination reduces the risk of investing in severe measures to combat the corona-pandemic which are not matched by others. The pressure for consensus in terms of membership and provision level is not an obstacle but is helpful in hedging against risk. Furthermore, introducing asymmetries in the game, which are clearly relevant to provide any meaningful advice for the corona pandemic, cooperation is needed in addition to coordination. Cooperation entails transfer payments which compensate those countries, which, in the absence of a treaty, would have lower autarky provision levels, in order to join the club, aiming at ambitious provision levels.

Fortunately, we have shown that theory (with or without mistakes) and experiments all highlight that cooperation institutions, such as treaties, have a role to play to foster cooperation. In a perfect world, self-enforcing institutions (treaties) could bring us to the best possible outcome. In our less than perfect world, self-enforcing institutions (treaties) can improve the situation and bring us closer to first-best.

Our theoretical results suggested that cooperation is particular successful and meaningful in a world of very asymmetric countries, such as in the classical North–South geopolitical context. In a recent opinion piece^19^, Robert E. Rubin and David Miliband, a former Treasury Secretary of the US and a former British Foreign Secretary, respectively, have argued that “providing support to developing countries is not only morally right, but also powerfully in the self-interest of richer states”. Our formal analysis provides a solid foundation for their opinion.

Our experimental results, together with a behavioral theory with mistakes also suggested that cooperative institutions are important even among similar countries, as optimal coordination cannot be taken for granted. This provides a rationale for regional cooperation, such as cooperation within the EU, if we assume that asymmetries are typically smaller than at the global scale.

In any case, the world should pursue efforts to create cooperative institutions around the corona-pandemic, to increase cooperation and to facilitate coordination on better outcomes. As discussed above, this is both necessary and likely to be successful, given the prevailing incentive structure. This is true for COVID-19 but also for future pandemics. Abandoning the WHO is probably not a step in the right direction, which does not exclude the possibilities for reforms.

## Footnotes

1 Most likely, the last pandemic of similar proportion was the so-called Spanish-flu that emerged in 1918. The world was at war and intercontinental travelling was rare.

2 Although the severity of this pandemic might recede in the coming months, there exists a large number of similar viruses that might provoke new pandemics. Thus, the issues discussed here will remain relevant in the years to come.

3 See “At Least 89 Vaccines Are Being Developed. It May Not Matter”, New York Times, April 29, 2020.

4 There also exists a weaker-link version where the smallest contribution has the largest marginal influence on utility, followed by the second smallest contribution, and so on. We ignore this complication for simplicity. See for instance Arce and Sandler (2001) and Cornes and Hartley (2007).

5 This game has two non-cooperative equilibria in pure strategies when focusing on symmetric players. In the first (“bad”) equilibrium, no country vaccinates any of its citizens. In the second equilibrium, all countries vaccinate their population above the level that ensures local eradication of the disease. Hence, in the second (“good”) equilibrium, the disease is eradicated globally. These two equilibria are probably too extreme to be relevant for COVID-19, not only because there is no vaccine currently available, but also because it is unlikely that all countries will be able to vaccinate a sufficient proportion of its population, due to a lack of sufficient resources. This is one reason that questions the assumption of symmetric players.

6 Different countries have adopted different strategies when confronted with COVID-19. Lockdowns have been more severe in Spain than in Sweden, which implies different economic costs. Also, the provision of ICU beds, including ventilators, for all patients in need has been very different across countries. The same will be true for measures of mass vaccination, once a vaccine will be available. At an abstract level, all these differences can be summarized in a single variable indicating the intensity and quality of virus control.

7 See “‘Europe Finally Got the Message’: Leaders Act Together on Stimulus”, New York Times, June 4, 2020.

8 See “Borders Won’t Protect Your Country from Coronavirus”, New York Times, July 6, 2020.

9 See https://www.who.int/news-room/feature-stories/detail/two-out-of-three-wild-poliovirus-strains-eradicated.

10 For details on the expected economic consequences of the COVID-19 pandemic, see International Monetary Fund (2020) and the Financial Times coronavirus economic tracker, available at https://www.ft.com/content/e5879009-f451-4a54-9374-03472f2c4085.

11 See “The WHO v Coronavirus: Why it Can't Handle the Pandemic”, The Guardian, April 10, 2020.

12 See “Sweden Stayed Open. A Deadly Month Shows the Risks”, New York Times, May 15, 2020.

13 See “How Texas Swaggered into a Coronavirus Disaster”, New York Times, June 28, 2020.

14 This literature is surveyed in Finus and Caparrós (2015), and the most influential papers are collected in this volume.

15 Similar notions of this transfer scheme have been developed by Eyckmans et al. (2012), Fuentes-Albero and Rubio (2010) and Weikard (2009).

16 See footnote 8.

17 Initial steps are the call by the WHO for ‘Access to COVID-19 Tools (ACT-Accelerator)’ see https://www.who.int/publications/m/item/access-to-covid-19-tools-(act)-accelerator, and the ‘Coronavirus Global Response’ promoted by the EU, see https://ec.europa.eu/commission/presscorner/detail/en/ip_20_797.

18 Known as agent quantal response equilibrium (McKelvey and Palfrey 1998).

19 See footnote 8.
